# Wavelet Scattering Transform for ECG Beat Classification

**DOI:** 10.1155/2020/3215681

**Published:** 2020-10-09

**Authors:** Zhishuai Liu, Guihua Yao, Qing Zhang, Junpu Zhang, Xueying Zeng

**Affiliations:** ^1^School of Mathematical Sciences, Ocean University of China, 238 Songling Road, Qingdao, Shandong 266100, China; ^2^Department of Cardiology, Qilu Hospital (Qingdao), Cheeloo College of Medicine, Shandong University, 758 Hefei Road, Qingdao, Shandong 266035, China

## Abstract

An electrocardiogram (ECG) records the electrical activity of the heart; it contains rich pathological information on cardiovascular diseases, such as arrhythmia. However, it is difficult to visually analyze ECG signals due to their complexity and nonlinearity. The wavelet scattering transform can generate translation-invariant and deformation-stable representations of ECG signals through cascades of wavelet convolutions with nonlinear modulus and averaging operators. We proposed a novel approach using wavelet scattering transform to automatically classify four categories of arrhythmia ECG heartbeats, namely, nonectopic (N), supraventricular ectopic (S), ventricular ectopic (V), and fusion (F) beats. In this study, the wavelet scattering transform extracted 8 time windows from each ECG heartbeat. Two dimensionality reduction methods, principal component analysis (PCA) and time window selection, were applied on the 8 time windows. These processed features were fed to the neural network (NN), probabilistic neural network (PNN), and *k*-nearest neighbour (KNN) classifiers for classification. The 4th time window in combination with KNN (*k* = 4) has achieved the optimal performance with an averaged accuracy, positive predictive value, sensitivity, and specificity of 99.3%, 99.6%, 99.5%, and 98.8%, respectively, using tenfold cross-validation. Thus, our proposed model is capable of highly accurate arrhythmia classification and will provide assistance to physicians in ECG interpretation.

## 1. Introduction

Cardiovascular diseases (CVDs) are the main causes of death globally. An estimated 17.9 million people died from CVDs in 2016, representing 31% of all global deaths [1]. There are many factors that lead to CVDs, including smoking and tobacco use, physical inactivity, poor dietary habit, overweight and obesity, etc. [2]. One broad group of complication of CVDs is arrhythmia, which expresses the electrical disfunction of the heart.

**Figure 1:**
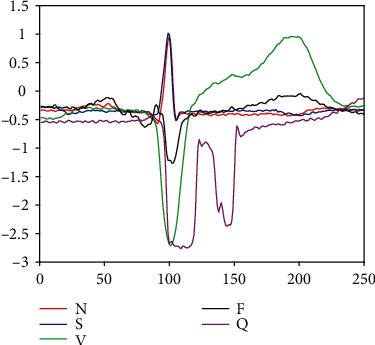
ECG signals for different arrhythmia categories.

An arrhythmia refers to the abnormal rate or rhythm of heartbeat. During an arrhythmia, the heart can beat too fast, too slowly, or with an irregular rhythm [3]. An electrocardiogram (ECG) monitors the electrical activity of the heart, and cardiac arrhythmias can be detected through any change in the morphological pattern over a recorded ECG waveform. There are many arrhythmia categories, and each contains different pathological information.  Figure 1 shows the patterns of ECG signals for different arrhythmia categories. It is of vital importance to accurately classify ECG signals into those categories in time. For cardiologists, relying on large amount of expertise and experience in their field, they visually observe the ECG waveform and obtain diagnostic results. However, this visual assessment may lead to subjective interpretations due to the presence of noise and minute morphological parameter values in ECG signals [4]. Moreover, it is also time-consuming and exhausting for cardiologists to interpret ECG signals, which may delay the best treatment opportunity for patients.

To address these drawbacks, various computer-aided diagnosis (CAD) systems have been developed recently. The CAD systems can be used as an adjunct tool for physicians in their interpretation of ECG signals to improve the accuracy and diagnostic speed. It plays an important role in the management of CVDs [5].  Table 1 summarizes some selected state-of-the-art studies of CAD systems. Most of them focused on conventional machine learning approaches. Feature extraction and classification are essential steps for these methods. The features extracted, including parametric and visual pattern features [6–8], from ECG signals and the classifiers designed for classification directly influence the performance of arrhythmia detection. Although some of these studies have achieved great classification performances, they might have two main drawbacks: firstly, they require a well-designed feature extractor and the features need to be manually optimized before feeding into classifiers; secondly, they usually suffer from overfitting. Moreover, few of these methods provided the confusion matrix recommended by the ANSI/AAMI EC57:1998 standard [9]. Hence, it is difficult to compare their classification performances on different arrhythmia categories in detail.

Since 2016, the methods based on deep learning approaches such as convolution neural network (CNN) have been proposed to identify abnormal ECG heartbeats including arrhythmias. Both of the feature extraction and classification are embedded together in the model. These methods have the ability to extract self-learn features [10]. However, they might have three main drawbacks: lack of strong theoretical support, requiring large amount of training data to achieve good performance, and consuming huge computational costs to train the model. Due to these drawbacks, one has to take a large number of numerical experiments to empirically conduct hyperparameter optimization as well as set up the optimal architecture, and the features extracted may be unexplainable in practical applications. Further, the performances of these methods remain to be improved.

The wavelet transform is an efficient tool for analyzing nonstationary ECG signals due to its time-frequency localization properties [11–13]. However, it is not invariant to translation. Recently, Mallat proposed a novel signal-processing method, the wavelet scattering transform, by cascading the wavelet transform with a nonlinear modulus and averaging operators [14]. The wavelet scattering transform can provide time and frequency resolutions, which is invariant to translation, stable to deformations, and preserves high frequency information for classification [15]. Moreover, Mallat characterized three properties that deep learning architectures possess for extracting useful features from data [16]: multiscale contractions, linearization of hierarchical symmetries, and sparse representation. The wavelet scattering transform also possesses these properties and, hence, has both advantages of conventional and deep learning approaches. It has achieved state-of-the-art performances in the tasks of art authentication, musical genre classification, audio recognition, and handwriting classification [17–20].

Motivated by the excellent property of wavelet scattering transform, we aim to explore the performance of the wavelet scattering transform in extracting the features from ECG signals for automated classification of arrhythmias. Specifically, we get data from the MIT-BIH Arrhythmia Database and classify the arrhythmias into four classes; more details are shown in Section 2. Then, we use wavelet scattering transform combined with some dimension reduction methods to extract features. Several existing classifiers, *k*-nearest neighbour (KNN), neural network (NN), and probabilistic neural network (PNN), are used to test the performances of the wavelet scattering transform on arrhythmia identification. In the end, our results are compared to some existing approaches listed in Table 1.

The paper is organized as follows: Section 2 introduces the database and data preprocessing methods.  Section 3 presents the wavelet scattering transform as well as its properties and introduces the classifiers used in this study.  Section 4 shows the detailed architecture and numerical experimental results, which are discussed in Section 5. We conclude the paper in Section 6.

## 2. Materials Used

In this section, we will briefly introduce the database that we used for ECG classification and describe our data preprocessing and augmentation methods.

### 2.1. MIT-BIH Database

**Figure 2:**
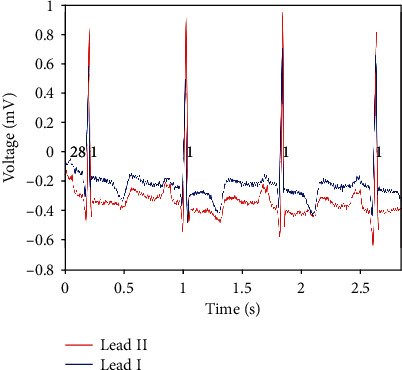
A fragment of record 100.

We used the MIT-BIH Arrhythmia Database [21] to train and test our method. This database is widely used for ECG classification and is publicly available. The MIT-BIH database contains 48 half-hour excerpts of two-channel ambulatory ECG recordings, obtained from 47 subjects studied by the BIH Arrhythmia Laboratory between 1975 and 1979 [22]. The recordings were digitized at 360 samples per second per channel with 11-bit resolution over a 10 mV range. These records were first annotated by at least two cardiologists independently. After reaching an agreement for all annotations, the agreed annotations were marked in a computer-readable format. The annotation for every beat on ECG includes the position of R-peak and the type of arrhythmia it belongs to. The database includes 15 types of arrhythmias such as ventricular premature, atrial premature, and atrial flutter.  Figure 2 shows a fragment of record 100. As shown in Figure 2, each record contains two leads, say, two channels of the ECG signal.

### 2.2. Data Preprocessing

According to the ANSI/AAMI EC57:1998 standard [9], the 15 types of arrhythmia beats can be classified into five categories including nonectopic (N) beats, supraventricular ectopic (S) beats, ventricular ectopic (V) beats, fusion (F) beats, and unknown (Q) beats.  Table 2 shows the subdivisions of these categories.

Complying with the ANSI/AAMI EC57:1998 recommended practice [9], we excluded 4 records which are from patients with pacemakers, because records containing paced beats do not retain sufficient signal quality. For the remaining records, only modified-lead II signals were used. Then, we detected the R-peak in each record to segment heartbeats. The R-peak detection algorithm is not the focus of our study, as many excellent algorithms have been proposed in literatures [11, 23]. Moreover, we directly used the raw data and no denoising technique was applied. Further details are available in [9].

A total of 100507 heartbeats were segmented from the 44 records. Each beat is 250 samples long, centered around the R-peak, containing 99 samples before the R-peak and 150 samples after the R-peak. Then, they were sorted into five categories according to their annotations.  Table 3 shows the number of heartbeats in each category. Similar to [6, 24, 25], the class Q was discarded since it is marginally represented (0.012%) in the database.  Figure 1 shows some segments in the considered four categories.

### 2.3. Data Augmentation

There are huge imbalances between the number of heartbeats in classes N, S, V, and F, which will lead to inferior classification performance [10, 26]. Following the data augmentation method in [26], we augmented the data by adding Gauss white noise with zero mean and 0.05 variance. Specifically, as class N has enough heartbeats, we randomly chose 90000 heartbeats from it and did not add noise. The number of beats in the remaining classes was increased to 90000 separately to match that in class N. Consequently, the augmented database includes 360000 heartbeats.

## 3. Methodology

In this section, we will present our methods for ECG classification. In Section 3.1, we describe the wavelet scattering transform that we used to learn the feature representation of ECG signals. We then introduce the used classifiers in Section 3.2.

### 3.1. Wavelet Scattering Transform

A wavelet scattering transform builds translation invariant, stable, and informative signal representations. It is stable to deformations and preserves class discriminability, which makes it particularly effective for classification. We refer to [17–20] for its excellent practical performance for classification.

We will follow the notations in [19]. Let *f*(*t*) be the signal under analysis. The low-pass filter *ϕ* and the wavelet function *ψ* are designed to build filters which cover the whole frequencies contained in the signal. Let *ϕ*_*J*_(*t*) be the low-pass filter that provides locally translation invariant descriptions of *f* at a predefined scale *T*. We denote by *Λ*_*k*_ the family of wavelet indices having an octave frequency resolution *Q*_*k*_. The multiscale high-pass filter banks {*ψ*_*j*_*k*__}_*j*_*k*_∈*Λ*_*k*__ can be constructed by dilating the wavelet *ψ*.

A wavelet scattering transform is implemented with a deep convolution network that iterates over traditional wavelet transform, nonlinear modulus, and averaging operators. The convolution *S*_0_*f*(*t*) = *f*⋆*ϕ*_*J*_(*t*) generates a locally translation invariant feature of *f*, but also results in the loss of high-frequency information. These lost high frequencies can be recovered by a wavelet modulus transform

(1) $$\begin{array}{c} \left|W_{1}\right|\;f={\left\{S_{0}f\left(t\right),\left|f⋆\psi_{j_{1}}\left(t\right)\right|\right\}}_{j_{1}\in \Lambda_{1}}. \end{array}$$

The first-order scattering coefficients are obtained by averaging the wavelet modulus coefficients with *ϕ*_*J*_:

(2) $$\begin{array}{c} S_{1}f\left(t\right)={\left\{\left|f⋆\psi_{j_{1}}\right|⋆\phi_{J}\left(t\right)\right\}}_{j_{1}\in \Lambda_{1}}. \end{array}$$

To recover the information lost by averaging, noting that *S*_1_*f*(*t*) can be seen as the low-frequency component of |*f*⋆*ψ*_*j*_1__|, we can extract complementary high-frequency coefficients by

(3) $$\begin{array}{c} \left|W_{2}\right|\left|f⋆\psi_{j_{1}}\right|={\left\{S_{1}f\left(t\right),\left|\left|f⋆\psi_{j_{1}}\right|⋆\psi_{j_{2}}\left(t\right)\right|\right\}}_{j_{2}\in \Lambda_{2}}. \end{array}$$

It further defines the second-order scattering coefficients

(4) $$\begin{array}{c} S_{2}f\left(t\right)={\left\{\left|\left|f⋆\psi_{j_{1}}\right|⋆\psi_{j_{2}}\right|⋆\phi_{J}\left(t\right)\right\}}_{j_{i}\in \Lambda_{i}},\;\;i=1,2. \end{array}$$

Iterating the above process defines wavelet modulus convolutions

(5) $$\begin{array}{c} U_{m}f\left(t\right)={\left\{\left|\left|\;|\;f⋆\psi_{j_{1}}\;|\;⋆\cdots \right|⋆\psi_{j_{m}}\right|\right\}}_{j_{i}\in \Lambda_{i}},\;i=1,2,\cdots ,m. \end{array}$$

Averaging *U*_*m*_*f*(*t*) with *ϕ*_*J*_ gives the *m*-th-order scattering coefficients

(6) $$\begin{array}{c} S_{m}f\left(t\right)={\left\{\left|\left|\;|\;f⋆\psi_{j_{1}}\;|\;⋆\cdots \right|⋆\psi_{j_{m}}\right|⋆\phi_{J}\left(t\right)\right\}}_{j_{i}\in \Lambda_{i}},\;i=1,2,\cdots ,m. \end{array}$$

**Figure 3:**
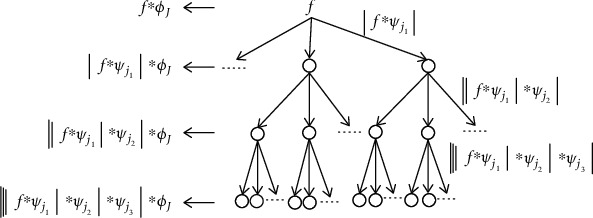
The tree view of wavelet scattering network.

This scattering process is illustrated in Figure 3. The final scattering matrix

(7) $$\begin{array}{c} Sf\left(t\right)={\left\{S_{m}f\left(t\right)\right\}}_{0\leq m\leq l}, \end{array}$$

aggregates scattering coefficients of all orders to describe the features of input signal, where *l*  is the maximal decomposition order.

The network is invariant to translations up to the invariance scale, which can be potentially large, due to the average operation determined by the low-pass filter *ϕ*_*J*_. As a property inherited from wavelet transform, the features *Sf*(*t*) are stable to local deformations. The scattering decomposition can capture subtle changes in amplitude and duration of ECG signals, which are hard to measure but reflect the condition of the heart. Therefore, we use the wavelet scattering network to produce robust representations of ECG heartbeats that minimize differences within one arrhythmia category while maintaining enough discriminability between different categories.

Though the structure of the wavelet scattering network is similar to CNN, they have two main differences: the filters are not learned but set in advance and the features are not only the output of the last convolution layer but also the combination of all those layers. It has been shown that the energy of scattering coefficients decreases rapidly as the layer level increases, with almost 99% of the energy contained in the first two layers [18, 19]. Therefore, we used a two-order scattering network to extract the features of ECG signals. This also reduces the computational complexity significantly.

### 3.2. Classifier

We next briefly introduce the used classifiers that combine features to predict the class membership of the ECG signal. We choose classifiers according to two criteria. First, the classifier must be widely used in existing literatures, such as NN, KNN, PNN, and support vector machine (SVM). Second, it must be capable of efficiently processing high dimension and large size training data. NN, KNN, and PNN satisfy both of the requirements, while SVM is ruled out for the low computational efficiency. Thus, we use NN, KNN, and PNN for classification in this work.

#### 3.2.1. Neural Network

The feedforward NN is the most widely used artificial neural network for classification [27, 28]. We set the architecture as follows. There are 75 neurons in the input layer, corresponding to the 75 dimensions of the feature vector extracted by wavelet scattering transform. Six hidden layers contain 70, 60, 45, 30, 20, and 10 neurons, respectively, and the first five hidden layers are activated by the ReLU function: *f*(*x*) = max(0, *x*). The output layer has 4 neurons, each of which represents an arrhythmia category and is activated by the Softmax function:

(8) $$\begin{array}{c} g{\left(z\right)}_{i}=\frac{\exp z_{i}}{\sum_{j=1}^{4}\exp z_{j}},\;i=1,\cdots ,4. \end{array}$$

We used the cross-entropy cost function [10] and employed error backpropagation algorithm to solve the weights. The Adam algorithm [29] was used to adaptively update the learning rate. We set the iteration number to 50 which is enough for training the network.

The above architecture was set up through trial and error. We have tried several combinations of different numbers of hidden layers, different activation functions, different numbers of neurons in each layer, different numbers of sample sizes in minibatch, and different epochs of parameter update, etc. Considering the computational cost and classification accuracy comprehensively, the network we present achieves the optimal performance compared to other tested architectures. Once the neural network was trained, all the testing data were fed into the network to measure its classification performance.

#### 3.2.2. Probabilistic Neural Network

The PNN [30] is widely used in classification and pattern recognition problems. In the PNN algorithm, the class probability of a new input data is estimated and the Bayesian rule is then employed to allocate the class with the highest posterior probability to new input data. The operations in a PNN are organized into a feedforward network with four layers: input layer, pattern layer, summation layer, and output layer. The input layer has the same number of neurons as the dimension of feature vector. Each neuron represents a predictor variable and feeds the values to each of the neurons in the pattern layer. The pattern layer contains one neuron for each sample in the training data. Each hidden neuron computes the Euclidean distance of the test sample from the neuron's center point. The summation layer has the same number of neurons as that of the categories of the input data. The weight coming out of a hidden neuron is fed only to the pattern neuron that corresponds to the hidden neuron's category. The output layer compares the weighted votes for each target category accumulated in the summation layer and uses the largest vote to predict the target category. PNN is more accurate than the multilayer neural network. It can approach the Bayesian optimal classification as long as the training data is enough. In this study, four layers in the trained PNN contain 75, 324000, 4, and 1 neurons, respectively.

#### 3.2.3. *k*-Nearest Neighbours

The KNN is a nonparametric method widely used for classification. The input consists of the *k* closest training samples in the feature space. An unlabeled data is classified by assigning the label which is most frequent among the *k* training samples nearest to that query data. The commonly used distance metric for KNN is the Euclidean distance. As for the selection of *k* values, we use the brute-force method. Specifically, *k* = 1, 2, 3, 4, 5 have been tested and *k* = 4 is the most appropriate value for the classification. Thus, we only present the results of *k* = 4 in Section 4.

## 4. Experimental Results

In this section, we will discuss the features extracted by scattering transform and our classification process. Specifically, two methods will be introduced for dimensionality reduction based on the pattern of features.

The wavelet scattering transform, PNN, and KNN classifiers were implemented by MATLAB 2018b. We used Python 3.7 to implement the NN classifier.

### 4.1. Feature Extraction

**Figure 4:**
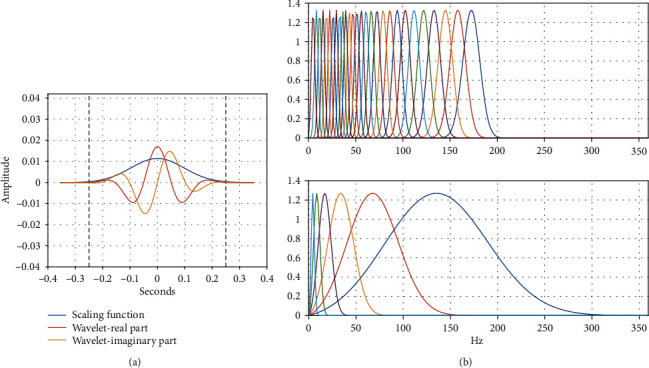
Wavelet filters. (a) The low pass filter with 0.5 s invariance scale. (b) The first filter bank with 8 wavelets per octave and the second filter bank with 1 wavelet per octave.

We used the Gabor wavelets to perform wavelet decomposition. The corresponding low-pass filter *ϕ* is a Gaussian function. We set the invariance scale to 0.5 second. The constructed wavelet scattering network includes two layers. We set *Q*_1_ = 8 and *Q*_2_ = 1 wavelets per octave at the first and second layers, respectively. We had tried other different settings for the invariance scale and wavelet octave resolution, but this architecture preserves the signal information best for classification.  Figure 4 shows the used Gabor wavelets and its low-pass filter *ϕ*_*J*_(*t*). Note that the coarsest-scale wavelet does not exceed the invariance scale determined by the time support of the low-pass filter *ϕ*_*J*_(*t*).

**Figure 5:**
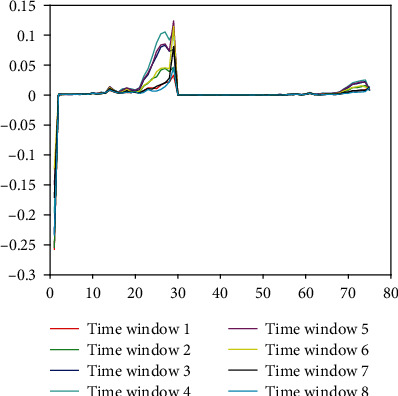
Scattering coefficients of 8 time windows for one ECG heartbeat.

The output of the wavelet scattering network forms a tensor with the size of 75 × 8 × 36000. Each slice of the tensor is the scattering coefficients of one ECG heartbeat. The scattering coefficients are critically downsampled in time based on the bandwidth of the low-pass filter, which results in 8 time windows for each of the 75 scattering paths. To obtain a data structure compatible with the used classifiers, we reshaped the tensor into a 2880000 × 75 matrix where each column and row corresponds to a scattering path and a time window, respectively. We obtained 2880000 rows because there are 8 time windows for each of the 360000 signals in the database.  Figure 5 shows the scattering coefficients of the 8 time windows for one ECG heartbeat.

### 4.2. Classification with NN

The NN classifier is capable of classification task for big data, so we used it to preliminarily test the classification performances of 8 time windows. For each heartbeat, we created labels to match the number of time windows. The decision for each time window was aggregated by majority vote to generate a label for the input ECG heartbeat.

We employed a 10-fold cross-validation [31]. Firstly, the 360000 ECG heartbeats were divided into 10 equal parts. Then, 90% of them were used to train the network, and the remaining 10% were used for testing. This process was repeated 10 times, and the overall performance was the averaged value over the 10 folds.

The AAMI has provided the standards and recommended practices for reporting performance results of automated arrhythmia detection algorithms [9]. We followed those practices so that the methods in this paper can be compared with those in Table 1. The positive predictive value (PPV), sensitivity (SEN), and specificity (SPEC) were used to measure the classification performances of our methods.  Table 4 presents the confusion matrix across 10 folds.  Table 5 presents the accuracy of each time window.

### 4.3. PCA and Time Window Selection

As shown in Figure 5, the 8 time windows are significantly correlated with each other.  Table 5 illustrates that the 3th, 4th, and 5th time windows have better discrimination than the others. We can also see from Figure 5 that these three time windows have larger amplitude and more fluctuations, which means they contain more and clearer details of ECG heartbeat, especially the 4th time window. In order to get better performance and reduce computational cost, we used two methods to reduce the redundancy of the 8 time windows.

- *Principal component analysis (PCA)*: PCA projects features in the directions of the highest variance to reduce the dimensionality of features [32]. The first few principal components can represent the most variability in features. The contribution rate of a principal component is the percentage of the total variability it represents. In this study, there are 8 time windows for each node in the scattering network. However, the 8 time windows have collinearity to some extent, which may lead to low classification performance. In order to remove the collinearity and generate more concise features, we used PCA to extract principal components of the 8 time windows for each node. The averaged contribution rate of the first and second principal components is approximately 84% and 15%, respectively. Hence, for each ECG heartbeat, we took the first principal component of 8 time windows as the new feature, which is a 75-dimensional vector with each dimension corresponding to a node.
- *Time window selection*: as described in Section 4.3, majority vote was used to predict the label for each testing ECG heartbeat. However, as shown in Table 5, the performance can be affected by those time windows with low accuracy. Moreover, the pathological information of ECG signals mainly concentrates around the R-peak, which has a very short duration. The discrimination between different arrhythmia categories may be involved in one particular time window. This motivates us to test the performance of each time window separately using different classifiers and find the time windows that generate the best classification results.

The NN classifier is capable of using any number of time windows as features. While limited by their computational ability, the PNN and KNN classifiers are suitable for the case of using one time window as features. To test the PCA method, we fed the first principal component of 8 time windows into the NN, PNN, and KNN classifiers, respectively. The confusion matrices across 10 folds are shown in Table 6. To test the time window selection method, we conducted two experiments. Firstly, we fed the 8 time windows into the NN, PNN, and KNN classifiers separately and found that the 4th time window generates the best performance. The confusion matrices are shown in Table 7. Secondly, we tried different time window combinations and classified them by the NN classifier and found that the combination of the 3th, 4th, and 5th time windows performs better than the others.  Table 8 presents the confusion matrix.

## 5. Discussion

In this section, we will discuss the classification results presented in Section 4.3 and compare our methods with those state-of-the-art studies.

*NN:* among all methods using the NN classifier, the one using the 4th time window as feature provides the maximum averaged ACC of 98.1% and averaged PPV, SEN, and SPEC of 99.3%, 98.2%, and 97.8%, respectively. Comparing Tables 4, 7, and 8, we can confirm that removing some time windows improves the classification performance. This indicates that there is some redundancy among the 8 time windows and the differences between the four categories (N, S, V, and F) are mainly reflected in the 3th, 4th, and 5th time windows. The performance of the 4th time window is close to that of the combination of the 3th, 4th, and 5th time windows. However, the training time of the latter is three times that of the single 4th time window. Moreover, the performance of the first principal component of 8 time windows is unsatisfactory, which is much worse than that of the 4th time window.

*PNN:* comparing Tables 6 and 7, the 4th time window and the first principal component provide almost the same results in combination with the PNN (spread = 0.01) classifier. The former is slightly better, yielding an averaged ACC, PPV, SEN, and SPEC of 99.0%, 98.7%, 99.9%, and 96.0%, respectively. We set the spread value by the brute-force method. The PNN classifiers with a spread value of 0.005, 0.01, 0.02, 0.03, 0.04, 0.1, and 1 have been tested, and the one with the spread value of 0.01 produces the best results. Table 7 shows that the SEN of supraventricular ectopic beats (SVEB) and ventricular ectopic beats (VEB) are 99.8% and 99.9%, respectively; it means that almost all the SVEB and VEB have been correctly detected. Therefore, the PNN classifier has excellent performance in classifying the SVEB and VEB, which should be paid more attention in clinical diagnosis.

*KNN:* the best performance of this work is achieved by KNN with *k* = 4 and using the 4th time window as the feature. The averaged ACC, PPV, SEN, and SPEC are 99.3%, 99.6%, 99.5%, and 98.8%, respectively, and are much better than those of the PCA features. However, this result only measures the performance in classifying normal (N) and abnormal (S, V, and F) ECG heartbeats. From Table 7, we can find that the PNN classifier performs better in classifying different arrhythmia categories, especially the VEB and SVEB.

Table 1 summarizes recent advances in automated classification of ECG beats using the MIT-BIH Arrhythmia Database. Only four of them have the same arrhythmia categories as this work, which are N, S, V, F, and Q. Martis et al. [32] used PCA on discrete cosine transform (DCT) coefficients computed from the segmented beats of ECG. The dimensionality-reduced features in combination with the KNN classifier yield the highest averaged ACC, SEN, and SPEC of 99.52%, 98.69%, and 99.91%, respectively. However, the confusion matrix was not provided in [32]. Li and Zhou [33] used wavelet packet entropy (WPE) and random forests (RF) to classify ECG signals into 5 categories; they obtained an ACC of 94.61%. Acharya et al. [26] used a 9-layer convolution neural network and achieved an averaged ACC, SEN, and SPEC of 93.47%, 96.01%, and 91.64%, respectively. Yang and Wei [6] combine parametric and visual pattern features and use KNN for classification. They obtain an overall ACC of 97.70%. The accuracies of V and S are not satisfying and reduce the overall accuracy significantly.

Table 9 summarizes the performances achieved by our methods. We can conclude from Tables 9 and 1 that the performance of this work is better than those state-of-the-art studies which classify ECG heartbeats into 5 categories (N, S, V, F, and Q). This demonstrates that wavelet scattering transform performs well in extracting the features of ECG heartbeats that minimize intraclass differences and maintain interclass discriminability. Moreover, the scattering coefficients in particular time windows contain more representative information for different categories than those in the other time windows. The dimensionality reduction of the 8 time windows eliminates the redundancy of features, which not only improves the classification performance but also reduces the computational cost. In this study, our results show that the scattering coefficients of the 4th time window contain sufficient information for the classification of arrhythmias.

## 6. Conclusion

In this study, we discussed the automated ECG classification using the nonlinear features extracted by wavelet scattering transform from ECG beats. Combined with proper classifiers, this study demonstrates that the wavelet scattering coefficients can be well utilized for classification and yield highly accurate classification results. Our results showed that the scattering coefficients of the 4th time window combined with the KNN classifier achieve the best performance. The averaged ACC, PPV, SEN, and SPEC are 99.3%, 99.6%, 99.5%, and 98.8%, respectively. In our future work, we will attempt to combine all time windows by a proper method and then feed them into a sparse classifier to improve the classification performance and reduce the computational cost. Moreover, all the work presented in Table 1 are patient independent, that is, ECG beats are collected from a patient pool and experiments are conducted without considering the autocorrelation of ECG beats from the same patient. Nascimento et al. [34] propose an innovation in the configuration of the structural cooccurrence matrix. It is also of great interest to expand the wavelet scattering transform to the patient-dependent classification of arrhythmias using ECG signals.

## Figures and Tables

**Table 1:** Selected automated ECG classification methods on the MIT-BIH Arrhythmia Database.

Author	Year	Method		Class	Performance
Conventional machine learning approaches					
Inan et al. [35]	2006	Feature extraction: classifier	WT and timing interval Neural network	3	ACC: 95.16%
Sayadi et al. [36]	2010	Feature extraction: classifier	Innovation sequence of EKF Bayesian filtering	2	ACC: 99.10%
					SEN: 98.77%
					SPEC: 97.47%
Martis et al. [32]	2012	Feature extraction: classifier	PCA SVM with RBF kernel	5	ACC: 98.11%
					SEN: 99.90%
					SPEC: 99.10%
Prasad et al. [37]	2013	Feature extraction: classifier	HOS+ICA KNN	3	ACC: 97.65%
					SEN: 98.75%
					SPEC: 99.53%
Martis et al. [38]	2013	Feature extraction: classifier	Cumulant+ICA KNN	3	ACC: 99.5%
					SEN: 100%
					SPEC: 99.22%
Martis et al. [7]	2013	Feature extraction: classifier	HOS+PCA LS-SVM	3	ACC: 93.48%
					SEN: 99.27%
					SPEC: 98.31%
Martis et al. [39]	2013	Feature extraction: classifier	Cumulant+PCA LS-SVM	5	ACC: 94.52%
					SEN: 98.61%
					SPEC: 98.41%
Martis et al. [32]	2012	Feature extraction: classifier	DCT+PCA SVM with RBF kernel	5	ACC: 99.52%
					SEN: 98.69%
					SPEC: 99.91%
Martis et al. [40]	2014	Feature extraction: classifier	ICA+DCT KNN	3	ACC: 99.45%
					SEN: 99.61%
					SPEC: 100%
Kaya and Pehlivan [41]	2015	Feature extraction: classifier	Genetic algorithms KNN	5	ACC: 99.69%
					SEN: 99.46%
					SPEC: 99.91%
Kaya and Pehlivan [8]	2015	Feature extraction: classifier	Time series+PCA KNN	5	ACC: 99.63%
					SEN: 99.29%
					SPEC: 99.89%
Li and Zhou [33]	2016	Feature extraction: classifier	WPE+RR RF	5	ACC: 94.61%
Mondjar-Guerra et al. [42]	2018	Feature extraction: classifier	Wavelets+LBP+HOS+several amplitude values RF	5	ACC: 94.5%
					SEN: 66.4%
					SPEC: 70.3%
Yang and Wei [6]	2020	Feature extraction: classifier	Combined parameter and visual pattern features of ECG morphology KNN	5	ACC: 97.70%
This work	2020	Feature extraction: classifier	WSN+the 4th time window PNN	4	ACC: 99.3%
					SEN: 99.5%
					SPEC: 98.8%

Deep learning approaches					
Martis et al. [40]	2014	9-layer deep convolution neural network		5	ACC: 93.47%
					SEN: 96.01%
					SPEC: 91.64%

ACC: accuracy; SEN: sensitivity; SPEC: specificity; WT: wavelet transform; EKF: extended Kalman filter; DCT: discrete cosine transform; DWT: discrete wavelet transform; HOS: higher order statistics; IC: independent component; ICA: independent component analysis; RR: RR intervals; WPE: wavelet packet entropy; LBP: local binary patterns; RF: random forest; LS-SVM: least square-support vector machine.

**Table 2:** MIT-BIH Arrhythmia Database beats classified as per ANSI/AAMI EC57:1998 standard [9].

N	S	V	F	Q
Normal	Atrial premature	Premature ventricular contraction	Fusion of ventricular and normal	Paced
Left bundle branch	Aberrant atrial			Fusion of paced and normal
Right bundle branch block	Nodal (junctional) premature	Ventricular escape		Unclassifiable
Atrial escape	Supraventricular premature			
Nodal (junctional) escape				

**Table 3:** The breakdown of five arrhythmia categories.

Class	Number of ECG heartbeats
N	90023
S	2758
V	6914
F	800
Q	12
Total	100507

**Table 4:** The confusion matrix for 8 time windows combined with the NN across 10 folds.

Original	Predicted						
	N	S	V	F	PPV (%)	SEN (%)	SPEC (%)
N	88681	369	737	213	90.9	98.5	96.7
S	4106	82994	2006	894	93.9	92.2	98.0
V	2669	2132	83701	1498	91.7	93.0	97.2
F	2124	2872	4843	80161	96.9	89.1	96.5

**Table 5:** The accuracy of each time window classified by the NN.

Time window	1	2	3	4	5	6	7	8
ACC (%)	88.9	90.3	92.2	92.8	92.2	91.2	89.8	87.3

**Table 6:** The confusion matrices for the first principal component combined with the NN, PNN, and KNN across 10 folds.

	Original	Predicted						
		N	S	V	F	PPV (%)	SEN (%)	SPEC (%)
NN	N	83508	2621	2799	1072	92.4	92.8	97.5
	S	3144	84182	1946	728	94.0	93.5	98.0
	V	1434	996	86100	1460	91.7	95.7	97.1
	F	2270	1803	3069	82858	96.2	92.1	97.4

PNN	N	86738	1225	1459	578	97.8	96.4	99.3
	S	1646	88085	214	55	98.6	97.9	99.5
	V	50	13	89797	140	98.1	99.8	99.4
	F	228	27	21	89724	99.1	99.7	99.9

KNN	N	87816	690	1091	403	96.5	97.6	98.8
	S	2418	86520	627	435	98.9	96.1	99.7
	V	156	62	89612	170	98.0	99.6	99.3
	F	572	186	112	89130	98.9	99.0	99.7

**Table 7:** The confusion matrices for the 4th time window combined with the NN, PNN, and KNN across 10 folds.

	Original	Predicted						
		N	S	V	F	PPV (%)	SEN (%)	SPEC (%)
NN	N	88146	811	789	254	93.8	97.9	97.8
	S	3181	85641	901	277	94.9	95.2	98.3
	V	1553	1323	85084	2040	94.4	94.5	98.1
	F	1108	2439	3343	83110	97.0	92.3	97.5

PNN	N	86415	2540	615	430	99.8	96.0	99.9
	S	171	89828	0	1	97.2	99.8	99.0
	V	1	71	89879	49	99.3	99.9	99.8
	F	0	1701	1645	86654	96.0	96.3	98.8

KNN	N	88915	644	281	160	98.5	98.8	99.5
	S	893	87177	1155	775	92.7	96.9	97.4
	V	496	4545	82247	2712	96.4	91.4	98.9
	F	0	3	0	89997	99.5	100	100

**Table 8:** The confusion matrix for the 3th, 4th, and 5th time windows combined with the NN across 10 folds.

Original	Predicted						
	N	S	V	F	PPV (%)	SEN (%)	SPEC (%)
N	88156	560	1042	242	95.3	98.0	98.4
S	2662	86180	721	437	96.3	95.8	98.8
V	909	933	86431	1727	94.6	96.0	98.2
F	794	1836	3163	84207	97.2	93.6	97.9

**Table 9:** Summary of classification results achieved by all the methods in this paper.

Feature extraction	Classifier	TP	TN	FP	FN	ACC (%)	PPV (%)	SEN (%)	SPEC (%)
WSN	NN	261101	88681	1319	8899	97.2	99.5	96.7	98.5
WSN+PCA	NN	263152	83508	6492	6848	96.3	97.6	97.5	92.3
	PNN	268076	86738	3262	1924	98.6	98.8	99.3	96.4
	KNN	266854	87816	2184	3146	98.5	99.2	98.8	97.6
WSN+the 3th, 4th, and 5th time window	NN	264158	88146	1854	5842	97.9	99.3	97.8	97.9
WSN+the 4th time window	NN	265091	87999	2001	4909	98.1	99.3	98.2	97.8
	PNN	269828	86415	3585	172	99.0	98.7	99.9	96.0
	KNN	268611	88915	1085	1389	99.3	99.6	99.5	98.8

TP: true positive; TN: true negative; FP: false positive; FN: false negative; WSN: wavelet scattering network.

## Data Availability

All the data utilized in our research can be accessed from http://ecg.mit.edu/dbinfo.html.

## References

[1] World Health Organization Cardiovascular diseases (CVDs). https://www.who.int/en/news-room/fact-sheets/detail/cardiovascular-diseases-(cvds).

[2] Benjamin E. J., Virani S. S., Callaway C. W. (2018). Heart disease and stroke statistics-2018 update: a report from the American Heart Association. *Circulation*.

[3] Nation Heart Lung and Blood Institute Arrhythmia. https://www.nhlbi.nih.gov/health-topics/arrhythmia.

[4] Martis R. J., Acharya U. R., Adeli H. (2014). Current methods in electrocardiogram characterization. *Computers in Biology and Medicine*.

[5] Hadhoud M. M. A., Eladawy M. I., Farag A. Computer aided diagnosis of cardiac arrhythmias.

[6] Yang H., Wei Z. (2020). Arrhythmia recognition and classification using combined parametric and visual pattern features of ECG morphology. *IEEE Access*.

[7] Martis R. J., Acharya U. R., Mandana K. M., Ray A. K., Chakraborty C. (2013). Cardiac decision making using higher order spectra. *Biomedical Signal Processing and Control*.

[8] Kaya Y., Pehlivan H. (2015). Classification of premature ventricular contraction in ECG. *International Journal of Advanced Computer Science and Applications*.

[9] Association for the Advancement of Medical Instrumentation (1998). Testing and reporting performance results of cardiac rhythm and ST segment measurement algorithms.

[10] Goodfellow I., Bengio Y., Courville A. (2016). *Deep Learning*.

[11] Li C., Zheng C., Tai C. (1995). Detection of ECG characteristic points using wavelet transforms. *IEEE Transactions on Biomedical Engineering*.

[12] Ince T., Kiranyaz S., Gabbouj M. (2009). A generic and robust system for automated patient-specific classification of ECG signals. *IEEE Transactions on Biomedical Engineering*.

[13] Martis R. J., Acharya U. R., Min L. C. (2013). ECG beat classification using PCA, LDA, ICA and discrete wavelet transform. *Biomedical Signal Processing and Control*.

[14] Mallat S. (2012). Group invariant scattering. *Communications on Pure and Applied Mathematics*.

[15] Bruna J., Mallat S. (2013). Invariant scattering convolution networks. *IEEE Transactions on Pattern Analysis and Machine Intelligence*.

[16] Mallat S. (2016). Understanding deep convolutional networks. *Philosophical Transactions of the Royal Society A: Mathematical, Physical and Engineering Sciences*.

[17] Leonarduzzi R., Liu H., Wang Y. (2018). Scattering transform and sparse linear classifiers for art authentication. *Signal Processing*.

[18] Bruna J., Mallat S. Classification with scattering operators.

[19] Andén J., Mallat S. Multiscale scattering for audio classification.

[20] Andén J., Mallat S. (2014). Deep scattering spectrum. *IEEE Transactions on Signal Processing*.

[21] Mark R., Moody G. MIT-BIH Arrhythmia Database. http://ecg.mit.edu/dbinfo.html.

[22] Moody G. B., Mark R. G. (2002). The impact of the MIT-BIH Arrhythmia Database. *IEEE Engineering in Medicine and Biology Magazine*.

[23] Pan J., Tompkins W. J. (1985). A real-time QRS detection algorithm. *IEEE Transactions on Biomedical Engineering*.

[24] Rahhal M. M. A., Bazi Y., AlHichri H., Alajlan N., Melgani F., Yager R. R. (2016). Deep learning approach for active classification of electrocardiogram signals. *Information Sciences*.

[25] Luo K., Li J. Q., Wang Z. G., Cuschieri A. (2017). Patient-specific deep architectural model for ECG classification. *Journal of Healthcare Engineering*.

[26] Acharya U. R., Oh S. L., Hagiwara Y. (2017). A deep convolutional neural network model to classify heartbeats. *Computers in Biology and Medicine*.

[27] Bishop C. M. (1995). *Neural Networks for Pattern Recognition*.

[28] Haykin S. (1994). *Neural Networks: A Comprehensive Foundation*.

[29] Kingma D. P., Ba J. (2014). Adam: a method for stochastic optimization. http://arxiv.org/abs/1412.6980.

[30] Specht D. F. (1990). Probabilistic neural networks. *Neural Networks*.

[31] Duda R. O., Hart P. E., Stork D. G. (2012). *Pattern Classification*.

[32] Martis R. J., Acharya U. R., Mandana K. M., Ray A. K., Chakraborty C. (2012). Application of principal component analysis to ECG signals for automated diagnosis of cardiac health. *Expert Systems with Applications*.

[33] Li T., Zhou M. (2016). ECG classification using wavelet packet entropy and random forests. *Entropy*.

[34] Nascimento N. M. M., Marinho L. B., Peixoto S. A., do Vale Madeiro J. P., de Albuquerque V. H. C., Filho P. P. R. (2020). Heart arrhythmia classification based on statistical moments and structural co-occurrence. *Circuits Systems and Signal Processing*.

[35] Inan O. T., Giovangrandi L., Kovacs G. T. A. (2006). Robust neural-network-based classification of premature ventricular contractions using wavelet transform and timing interval features. *IEEE Transactions on Biomedical Engineering*.

[36] Sayadi O., Shamsollahi M. B., Clifford G. D. (2010). Robust detection of premature ventricular contractions using a wave-based Bayesian framework. *IEEE Transactions on Biomedical Engineering*.

[37] Prasad H., Martis R. J., Acharya U. R., Min L. C., Suri J. S. Application of higher order spectra for accurate delineation of atrial arrhythmia.

[38] Martis R. J., Acharya U. R., Prasad H., Chua C. K., Lim C. M., Suri J. S. (2013). Application of higher order statistics for atrial arrhythmia classification. *Biomedical Signal Processing and Control*.

[39] Martis R. J., Acharya U. R., Lim C. M., Mandana K. M., Ray A. K., Chakraborty C. (2013). Application of higher order cumulant features for cardiac health diagnosis using ECG signals. *International Journal of Neural Systems*.

[40] Martis R. J., Acharya U. R., Adeli H. (2014). Computer aided diagnosis of atrial arrhythmia using dimensionality reduction methods on transform domain representation. *Biomedical Signal Processing and Control*.

[41] Kaya Y., Pehlivan H. Feature selection using genetic algorithms for premature ventricular contraction classification.

[42] Mondjar-Guerra V., Novo J., Rouco J., Penedo M. G., Ortega M. (2019). Heartbeat classification fusing temporal and morphological information of ECGs via ensemble of classifiers. *Biomedical Signal Processing and Control*.

